# A Comparative Genomic Survey Provides Novel Insights into Molecular Evolution of l-Aromatic Amino Acid Decarboxylase in Vertebrates

**DOI:** 10.3390/molecules23040917

**Published:** 2018-04-16

**Authors:** Yanping Li, Yunyun Lv, Chao Bian, Xinxin You, Li Deng, Qiong Shi

**Affiliations:** 1 Shenzhen Key Lab of Marine Genomics, Guangdong Provincial Key Lab of Molecular Breeding in Marine Economic Animals, BGI Academy of Marine Sciences, BGI Marine, BGI, Shenzhen 518083, China; liyanping@genomics.cn (Y.L.); lvyunyun@genomics.cn (Y.L.); bianchao@genomics.cn (C.B.); youxinxin@genomics.cn (X.Y.); 2 BGI Education Center, University of Chinese Academy of Sciences, Shenzhen 518083, China; 3 Laboratory of Aquatic Genomics, College of Life Sciences and Oceanography, Shenzhen University, Shenzhen 518060, China; lideng03@szu.edu.cn

**Keywords:** l-aromatic amino acid decarboxylase (AAAD), melatonin biosynthesis, molecular evolution, vertebrate, pseudogene

## Abstract

Melatonin is a pleiotropic molecule with various important physiological roles in vertebrates. l-aromatic amino acid decarboxylase (AAAD) is the second enzyme for melatonin synthesis. By far, a clear-cut gene function of AAAD in the biosynthesis of melatonin has been unclear in vertebrates. Here, we provide novel insights into the evolution of AAAD based on 77 vertebrate genomes. According to our genome-wide alignments, we extracted a total of 151 *aaad* nucleotide sequences. A phylogenetic tree was constructed on the basis of these sequences and corresponding protein alignments, indicating that tetrapods and diploid bony fish genomes contained one *aaad* gene and a new *aaad*-like gene, which formed a novel AAAD family. However, in tetraploid teleosts, there were two copies of the *aaad* gene due to whole genome duplication. A subsequent synteny analysis investigated 81 *aaad* sequences and revealed their collinearity and systematic evolution. Interestingly, we discovered that platypus (*Ornithorhynchus anatinus)*, Atlantic cod (*Guadus morhua*), Mexican tetra (*Astyanax mexicanus*), and a *Sinocyclocheilus* cavefish (*S. anshuiensis*) have long evolutionary branches in the phylogenetic topology. We also performed pseudogene identification and selection pressure analysis; however, the results revealed a deletion of 37 amino acids in Atlantic cod and premature stop codons in the cave-restricted *S. anshuiensis* and *A. mexicanus*, suggesting weakening or disappearing rhythms in these cavefishes. Selective pressure analysis of *aaad* between platypus and other tetrapods showed that rates of nonsynonymous (*Ka*) and synonymous (*Ks*) substitutions were higher when comparing the platypus to other representative tetrapods, indicating that, in this semiaquatic mammal, the *aaad* gene experienced selection during the process of evolution. In summary, our current work provides novel insights into *aaad* genes in vertebrates from a genome-wide view.

## 1. Introduction

Molecular activity and individual behavior are adjusted by a biological process related to circadian rhythms, which synchronize with the local environment through various external clues, such as light, darkness, temperature, water salinity, and food availability. Cycles of day and night have significant impacts among these clues. Composed of a clock machinery, the circadian rhythm system contains unique elements, by which light enters organisms to transform into hormonal signals. In turn, the internal clock drives rhythmic signal output. Melatonin, *N*-acetyl-5-methoxy tryptamine, acts as one major output of vertebrate circadian clocks and sends rhythmic signals back to organisms [1].

Melatonin was isolated from the bovine pineal gland by Dr. Aaron B. Lerner and his colleagues in 1958 [2]. Subsequently, it has been detected in many extra-pineal organs, including the retina, Harderian gland, skin, gut, ovary, testis, bone marrow, and immune system cells of vertebrates [3,4,5,6,7]. However, despite widespread existence of melatonin in these organs, the pineal gland and retina represent the two most important organs with the highest content of melatonin. Previous studies reported that melatonin has been involved in multiple adjustments of circadian rhythms, including sleep-wake timing, appetite regulation, seasonal reproduction, blood pressure regulation, enhanced immunity, increased antioxidant capacity, and elimination of free radicals [8,9]. Melatonin in adjusting the circadian rhythms mainly reflects its secretion levels at different time points. In vertebrates, it is much conserved of melatonin secretion in a daily pattern. Under normal physiological conditions, the cycle of melatonin secretion is synchronized with photoperiod, showing a distinct circadian rhythm, with a peak in the middle of night and gradual decrease towards morning in blood and cerebrospinal fluid [10]. Many animals also use variation in duration of melatonin production each day as a seasonal clock.

**Figure 1:**
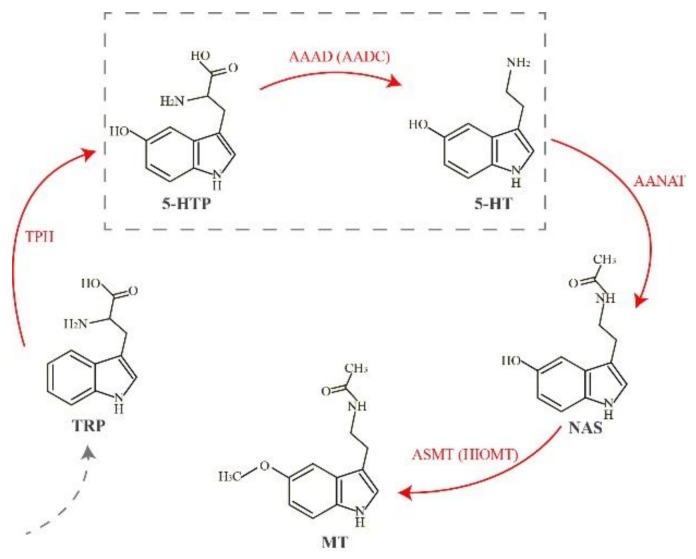
The classical pathway of melatonin biosynthesis in vertebrates. Red arrows emphasize the four melatonin synthetases. The dashed box marks the main step involved in our present study. Abbreviations: AAAD, l-aromatic amino acid decarboxylase; AANAT, aralkylamine *N*-acetyltransferase; ASMT: acetylserotonin-*O*-methyltransferase; 5-HT, 5-hydroxytryptamine; 5-HTP, 5-hydroxytryptophan; MT, melatonin; NAS: *N*-acetylserotonin; TPH, tryptophan hydroxylase; TRP, tryptophan.

Melatonin acts as a pleiotropic regulator molecule. A lack of melatonin synthetases or melatonin receptors can lead to a decreased level of melatonin and may eventually initiate many dysfunctions. In vertebrates, the melatonin biosynthesis pathway is a well-characterized cascade starting from the initial precursor tryptophan (Trp), and sequentially involves four enzyme-catalyzed reactions through hydroxylation, decarboxylation, acetylation, and methylation (Figure 1). In the first step, Trp is transformed into 5-hydroxytryptophan (5-HTP) through tryptophan hydroxylase (TPH, EC 1.14.16.4), which is encoded by the tryptophan hydroxylase 1 gene (*Tph1*) [11]. Subsequently, 5-HTP is catalyzed to serotonin (5-hydroxytryptamine, 5-HT) by l-aromatic amino acid decarboxylase (AAAD, which is also known as AADC, EC 4.1.1.28) [12]. In the third reaction, aralkylamine *N*-acetyltransferase (AANAT, EC 2.3.1.87) catalyzes serotonin to *N*-acetylserotonin (NAS) [13]. Finally, NAS is transformed by acetylserotonin-*O*-methyltransferase (ASMT, previously known as HIOMT; EC 2.1.1.4) to produce melatonin (MT) [14].

AAAD (or AADC), also known as tryptophan decarboxylase or 5-hydroxytryptophan decarboxylase, is a dimeric enzyme. It can make l-Dopa convert to dopamine; thus, AAAD is also named as DOPA decarboxylase (DDC). AAAD is mainly considered as a decarboxylizing enzyme, with a vital responsibility to synthesize neurotransmitters, including dopamine and serotonin [15]. In the pathway of melatonin synthesis, AAAD can decarboxylate 5-HTP to 5-HT. Moreover, AAAD is also involved in several neurological diseases, such as Parkinson’s disease, depression, and schizophrenia. When people are treated with l-DOPA for Parkinson’s disease, or treated with 5-HTP to deal with depression or dysthymia, AAAD has become the rate-limiting protein in both the dopamine and serotonin synthetic pathways. People with AAAD deficiency showed compromised development, especially in motor functions [16]. Thus, investigations on how AAAD affects Parkinson’s disease in mammals have been very active recently [16,17,18]. Nevertheless, according to a recent research, researchers suggested that AAAD activity may play an important role in the regulation of melatonin synthesis via controlling the availability of 5-HT [19]. However, a clear-cut gene function of AAAD in the biosynthesis of melatonin has been unclear in vertebrates so far. With the development of high-throughput sequencing and availability of genome data from many vertebrates, we can explore *aaad* genes based on the accumulated genomic data. For a better understanding of the melatonin synthesis pathway in vertebrates, we sought to explore molecular evolution of AAAD in vertebrates.

In the present research, we focused on 77 genomes from vertebrate species. In teleosts, several species with special living habitats such as cavefishes (Mexican tetra *Astyanax mexicanus* and *Sinocyclocheilus anshuiensis* (Sa)), amphibious mudskippers (blue-spotted *Boleophthalmus pectinirostris* (BP) and giant-fin *Periophthalmus magnuspinnatus* (PM)), and tetraploid fishes (Atlantic salmon *Salmo salar*, Rainbow trout *Oncorhynchus mykiss*) have been emphasized for examination. We determined presence or absence of *aaad* genes in vertebrates, as well as sequence differences across species for construction of a phylogenetic tree based on these extracted sequences. We further implemented a synteny analysis and mapped the outcome into another phylogenetic tree. Subsequently, we identified pseudogenes in fishes with special habitats, such as deep-sea Atlantic cod (*Guadus morhua*) and cave-restricted Mexican tetra and Sa. Selective pressure between the semiaquatic cave-dwelling platypus (*Ornithorhynchus anatinus*) and other selected tetrapods was also compared. We finally attempted to answer the following three core questions: (1) presence or absence of species-specific *aaad* gene(s) and sequence differences among vertebrates; (2) existence or extinction of different evolutionary models of AAAD between tetrapods and teleosts, especially for the cavefishes and tetraploid teleosts; and (3) how *aaad* genes impact on the circadian rhythms of vertebrates.

## 2. Results

### 2.1. Collection of AAAD, Copy Number Variation, and Phylogenetic Relationships in Vertebrates

We collected a total of 151 *aaad* nucleotide sequences from 77 genomes of vertebrates, including 10 mammals, 23 birds, 10 reptiles, two amphibians, and 32 fish species (Table 1). Accession numbers of these genomes are provided in Table S1. Corresponding protein sequences of AAADs contained around 480 amino acids (aa) each, which is consistent with the data reported by other investigations [20,21].

In tetrapods, from each of almost all the examined species, we obtained two *aaad* sequences. However, in platypus and garter snake (*Thamnophis sirtalis*), we only identified one *aaad* sequence. Similarly, most teleost species were also proved with two *aaad* sequences; while for tetraploid teleosts, like Atlantic salmon, rainbow trout, Sg (*Sinocyclocheilus grahami*), and Sr (*S. rhinocerous*), two to three *aaad* sequences were searched out. Nevertheless, Atlantic cod, Mexican tetra, red-bellied piranha (*Pygocentrus nattereri*), and elephant shark (*Callorhynchus milii*) were identified with one *aaad* sequence in each species (see more details in Table S2). *aaad* genes predicted by us in golden-line barbel fishes (such as Sr) and mudskippers were also supported by transcriptome assemblies (see more information in Section 4.1).

**Figure 2:**
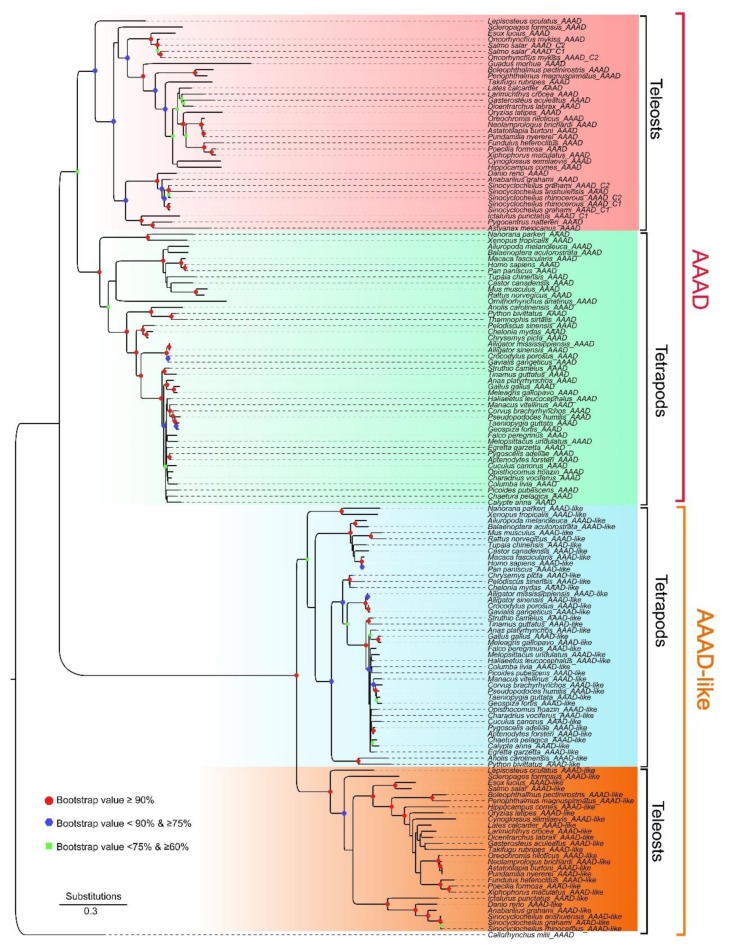
A phylogenetic tree based on 151 extracted nucleotide sequences in vertebrates. Red and orange shaded brackets marked AAAD and AAAD-like lineages, respectively. Bootstraps higher than 60% are displayed. Red dots indicate bootstraps ≥90%; blue hexagons represent bootstraps ≥75% and <90%; green squares stand for bootstraps ≥60% and <75%.

Furthermore, we constructed a phylogenetic tree based on the extracted 151 nucleotide sequences with elephant shark as the outgroup (Figure 2). It appears that the topology of this evolutionary tree was divided into two major lineages, and both were further split into two sub-lineages for tetrapods and teleosts. All AAADs from the tetrapods formed a sister group with the teleost species (the upper part in Figure 2), suggesting stability of the topology. Based on the phylogenetic results and combined with the reported structure of human AAAD (accession number 3RBF in the Protein Data Bank), we tentatively named the two lineages as AAAD and AAAD-like (the lower part in Figure 2), respectively.

### 2.2. Protein Structure Variations of AAADs and AAAD-Likes

**Figure 3:**
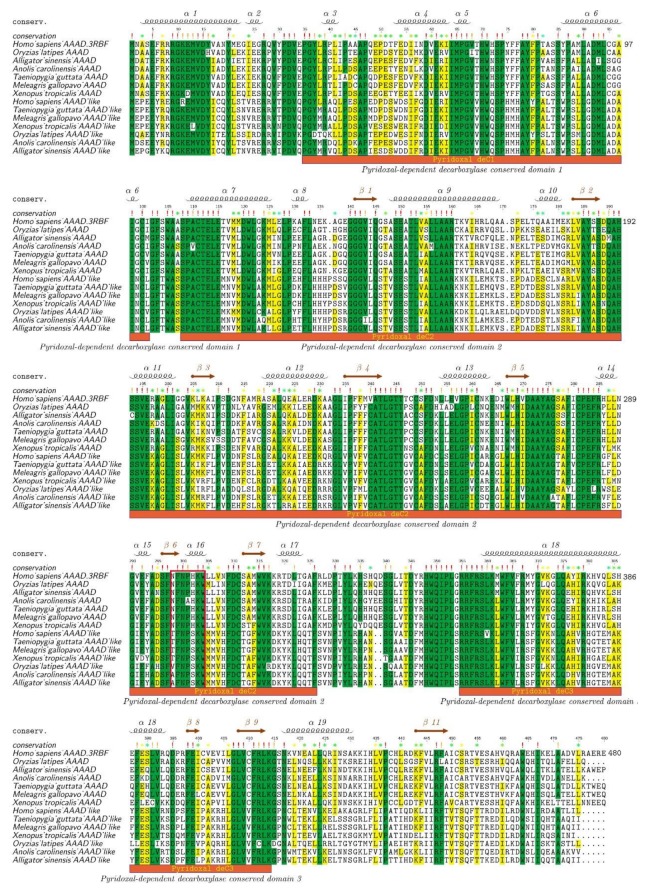
Alignment of protein sequences between AAADs and AAAD-likes from representative vertebrate species. The human AAAD sequence and structural template (PDB 3RBF) were employed as the reference for comparison and numbering. Sequence alignments were realized in Mega and colorized by TEXshade. Secondary structures include alpha helix (α) and beta strand (β). The special heptapeptide residue region was marked within the red box. Color codes for conservation track ranged from green (the most conserved) to yellow (the least conserved).

To provide further evidence for the division of AAAD and AAAD-like, we chose representative species from both lineages to perform alignment of protein sequences (Figure 3). Our data revealed that many residues in AAADs and AAAD-likes were highly conserved in vertebrates. However, some variations were still present in the residue peptide regions between AAADs and AAAD-likes. Previous researchers have identified a heptapeptide residue (Asn-Phe-Asn-Pro-His-Lys-Trp) for binding pyridoxal phosphate, and this region was a possible cofactor binding site [22]. Interestingly, we also observed this heptapeptide residue region (298th–304th aa; the red box in Figure 3) in our examined fish AAAD sequences.

According to the protein alignments, we can clearly tell the differences among the residues of 298th–302nd aa between AAADs and AAAD-likes. The 298th aa of AAAD was Asn (N), while it was Thr (T)/Val (V)/Ala (A) in AAAD-likes. The 302nd aa of AAAD was His (H), while it was Ser (S) in AAAD-likes. Based on the human template of 3RBF [23], the 298th aa participates in the formation of the α-helix, and the 302nd aa is involved in forming the β-turn. Some other variation sites between AAADs and AAAD-likes are also marked out in Figure 3. Combining the phylogenetic tree and protein alignments, we propose that a lineage with the reported seven-residue peptide region for bindings of pyridoxal phosphate is AAAD, while another lineage with changes at this heptapeptide region is named as AAAD-like. All in all, protein alignments showed a high conservation of the AAAD family (including AAADs and AAAD-likes) in vertebrates.

### 2.3. Synteny and Phylogeny Analysis

**Figure 4:**
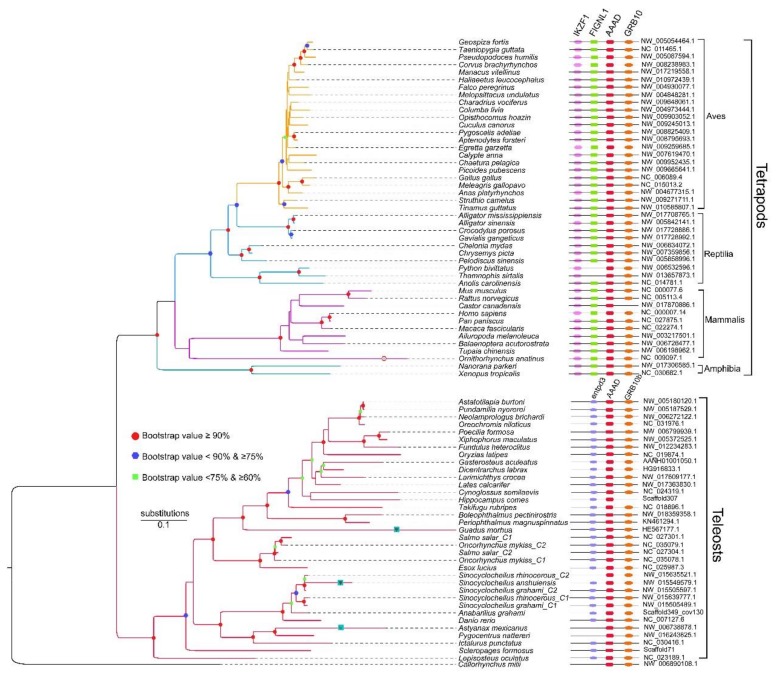
A phylogenetic tree based on 81 AAAD synteny sequences. Shapes in the nodes indicate bootstraps obtained in the PhyML reconstruction, and only the values higher than 60% are shown. Red dots indicate bootstraps ≥90%; blue hexagons represent bootstraps ≥75% & <90%; green squares stand for bootstraps ≥60% and <75%. “ψ” stands for a possible pseudogene with missing exon(s), or codon frameshift(s) or premature stop codon(s). Note the pink hexagon for the longest branch of platypus (*Ornithorhynchus anatinus*) in the tetrapod lineage.

We performed additional synteny analysis to distinguish AAADs and AAAD-likes. Firstly, we investigated the upstream and downstream of *aaad* genes in tetrapods and teleosts, which demonstrated that synteny genes were different between tetrapods and teleosts (Figure 4). We observed that three conserved genes, *IKZF1* (Ikaros family zinc finger 1), *FIGNL1* (fidgetin like 1) and *Grb10* (growth factor receptor-bound protein 10), within the neighboring positions of *aaad*s in tetrapods, while all these synteny genes could not be identified in teleosts; however, two other genes, *entpd3* (ectonucleoside triphosphate diphosphohydrolase 3) and *Grb10b*, were localized near the downstream and upstream of *aaad* in teleosts.

Subsequently, we performed a comparative synteny analysis between tetrapods and teleosts. In tetrapods, all *aaad* genes shared a conserved suite of genes around them, although the Burmese python (*Python bivittatus*) and garter snake (*Thamnophis sirtalis*) may display gene loss. Similarly, all *aaad* genes in teleosts also displayed conservation with the neighboring genes, but the tiger-tailed seahorse (*Hippocampus comes*) lose *Grb10b*; Mexican tetra, red-bellied piranha, elephant shark, and one copy in Sr possibly lose *entpd3* (see more details in the right comparison of Figure 4).

We further extracted synteny sequences from all the above-mentioned 151 sequences, and only 81 synteny sequences were identified (Figure 4). Interestingly, all these 81 synteny sequences were in full accord with the AAAD lineage in the phylogeny tree that was constructed with the 151 sequences (Figure 2). Our synteny results supported the rationality to classify the two lineages as AAAD and AAAD-like. To resolve genetic underpinnings of AAAD in vertebrates, we further constructed another phylogenetic tree based on these 81 synteny sequences. Corresponding topology (the left section of Figure 4) was also divided into two clades, in which tetrapod species formed a sister group with teleosts. In the clade of tetrapods, Aves have a close relationship with reptiles, and species from these two classes formed a sister group with mammals, while amphibians have the farthest relationship with other species in tetrapods. Moreover, we observed that platypus, Atlantic cod, Mexican tetra, and Sa have long evolutionary branches (left in Figure 4), which indicated higher evolutionary rates or possible pseudogenes.

### 2.4. Pseudogene Identification and Prediction of Three-Dimensional (3D) AAAD Structures

**Figure 5:**
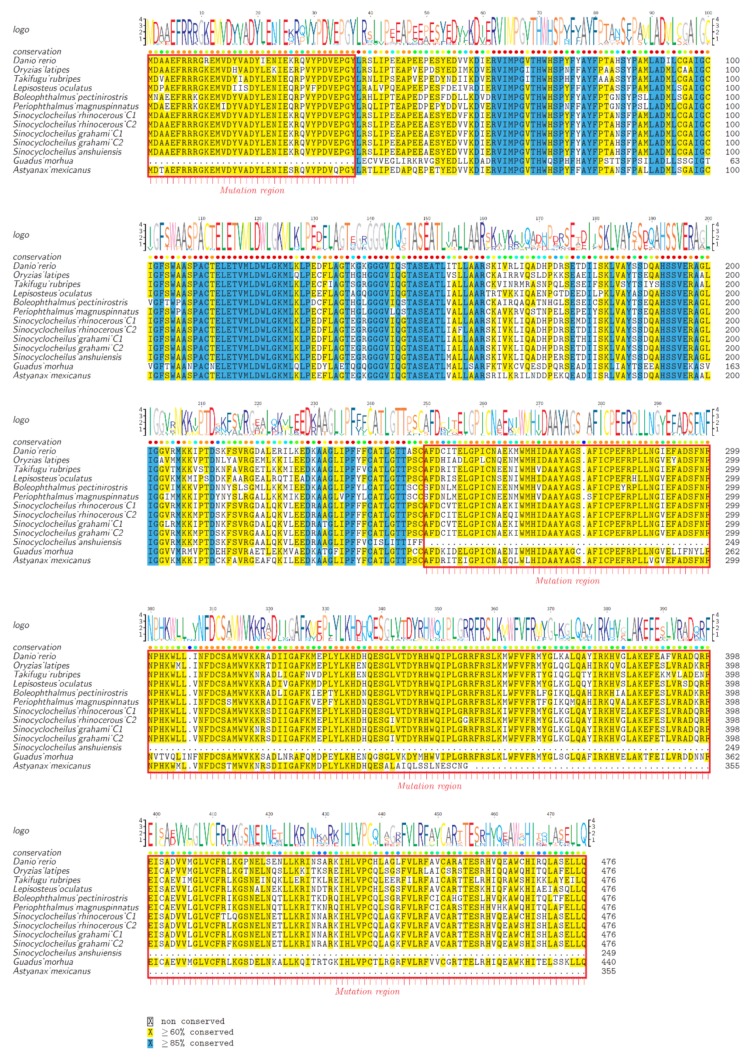
Pseudogene identification in *aaad*s from several representative teleost species. The analysis was conducted in Mega and colorized by TEXshade. Missing exons and premature stop codons are marked within the red boxes.

To test whether AAADs from cavefishes (Sa and Mexican tetra) or deep-sea Atlantic cod show pseudogenization, we chose and compared AAAD protein sequences from amphibious teleost fishes, cave-restricted fishes, deep-sea fishes, and model fishes. Our results demonstrated a deletion of 37 amino acids in Atlantic cod AAAD, with a premature stop at 250th and 356th aa in cave-restricted Sa and Mexican tetra, respectively (red boxes in Figure 5). This pseudogenization suggests weakening or disappearing rhythms in these fishes.

**Figure 6:**
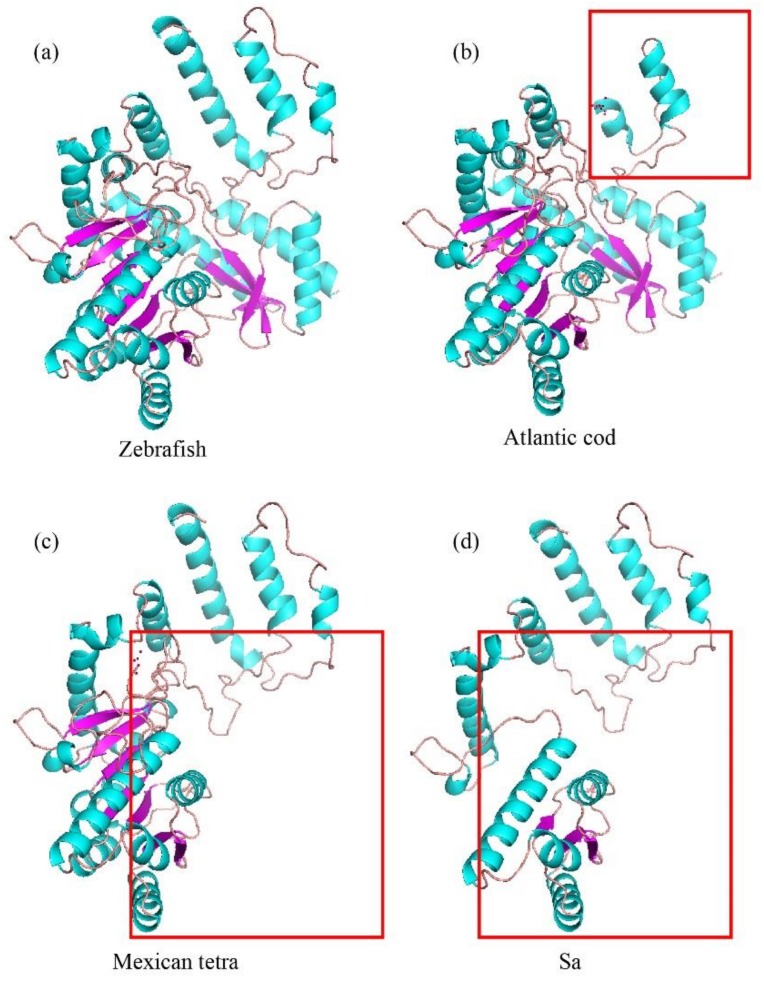
Predicted 3D structures of AAADs structures among zebraﬁsh (**a**), Atlantic cod (**b**), Mexican tetra (**c**), and Sa (**d**). Red boxes indicate the amino-terminal or carboxyl-terminal deletions.

In the comparisons of AAAD 3D structures among zebrafish (*Danio rerio*), Atlantic cod, Mexican tetra, and Sa (Figure 6), we observed the similarity between zebrafish and human AAADs (Figure 6a); however, Atlantic cod presented a lack in the N-terminal (red rectangle in Figure 6b). In contrast, the cave-restricted Mexican tetra and Sa displayed a shortage of the C-terminal (red rectangles in Figure 6c,d). These differences reflect remarkable structural variations in AAAD proteins among these fishes.

### 2.5. Substitution Changes

**Figure 7:**
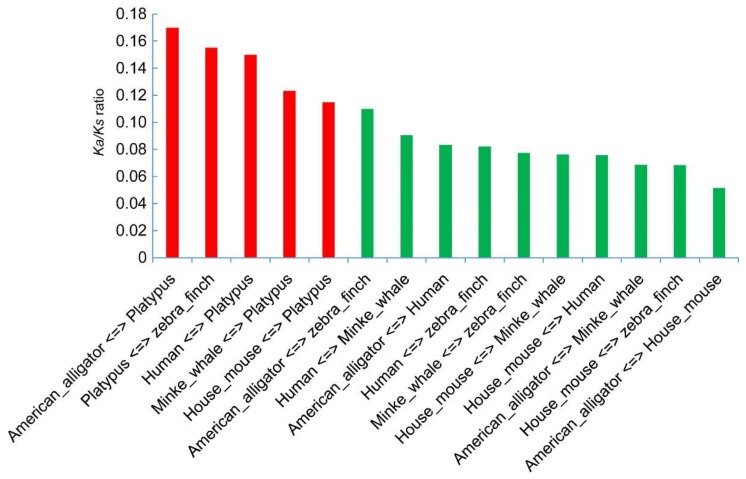
Ratios of non-synonymous and synonymous substitutions (*Ka*/*Ks*). They were estimated with DnaSP 5.0 based on AAAD proteins from six tetrapod species. Fifteen pairs of combinations are sorted from left to right for comparison.

The ratio between *Ka* (nonsynonymous) and *Ks* (synonymous) substitutions is an effective indicator that has been widely accepted to measure the selective pressure in sequence evolution. Usually, the *Ka*/*Ks* ratio more than 1 indicates evidence for a positive selection, and less than 1 means a negative selection [24,25]. Among the calculations on the fifteen pairs from the six organisms (Figure 7), all *Ka/Ks* values were much less than 1, indicating that negative selection during evolution affected the *aaad* changes in tetrapods (Table S3). However, the *Ka*/*Ks* ratios present relatively higher values in the pairs containing platypus (red bars in Figure 7) than those pairs without platypus, suggesting that *aaad* gene in platypus may have experienced a natural selection for the burrowing habitats during its process of evolution. 

## 3. Discussion

To our knowledge, this study is the first comprehensive investigation on AAAD evolution in vertebrates. We surveyed many facets of *aaad* family genes (including both *aaad*s and *aaad*-likes), and provided novel insights into diversity and structural variations of AAADs in vertebrates from a genomics view. For the first time, we confirmed the existence of one *aaad* and a new *aaad*-like gene in the genomes of tetrapods and diploid bony fish. For tetraploid teleosts, however, two copies of *aaad* genes were identified from their genome sequences, which may be due to a whole genome duplication. Pseudogene identification proved a deletion or premature stop in deep-sea Atlantic cod and cave-restricted Sa and Mexican tetra, suggesting weakening or disappearing rhythms in these fishes. Moreover, *Ka*/*Ks* ratios of *aaad* genes between platypus and other tetrapods suggested a potential positive selection in the platypus during its evolution.

### 3.1. Possible Reasons for Copy Number Variations Among Vertebrates

As we know, the common ancestor of early vertebrates underwent two rounds of whole genome duplication (WGD) [26,27]. Specifically, one round of WGD occurred before the split of Agnatha-Gnatostoma, and another WGD happened before the Chrondrichthyes-Osteichthyes split. After the Actinopterygian-Sarcopterygian split, a third round of WGD that is specific to teleosts subsequently occurred [28,29]. Fishes from families of Acipenseridae, Catostomidae, Cobitidae, Cyprininae, and Salmonidae even went through a fourth round of WGD [30]. Almost all species of tetrapods and diploid fishes in our present study had one copy of *aaad* and *aaad*-like genes, while tetraploid teleosts, like Atlantic salmon, Rainbow trout, Sg, and Sr, had two copies of the *aaad* gene. Atlantic salmon and rainbow trout experienced salmonid-specific genome duplication, which led to the appearance of two copies of the *aaad* gene in their genomes. However, in another tetraploid fish, Amazon molly (*Poecilia formosa*), one copy of the *aaad* gene was lost, since we extracted only one *aaad* copy in its genome. This is similar to our previous study on molecular evolution of AANAT [31], in which one copy of both *aanat1a* and *aanat1b* were also lost in the Amazon molly.

According to our results, we proposed that the main reasons for copy number variations of AAAD in vertebrates were caused by a combination of WGD and gene loss, just like the cases of AANAT [31,32] and ASMT [33], the last two rate-limiting enzymes for melatonin biosynthesis (Figure 1). Due to selective loss of genes, the number of copies between diploid and tetraploid does not always correspond to one-to-two. For *aaad*-like genes, most examined species in our present study have one copy, while in platypus and garter snake it is lost. This phenomenon is more common in teleosts, and we did not find *aaad*-like genes in Atlantic cod, Mexican tetra, rainbow trout, red-bellied piranha, and elephant shark. Hence, we deduce that the *aaad*-like genes may have evolved after the WGD event.

Moreover, our synteny analysis results revealed that *aaad* and *aaad*-like genes localize on different chromosomes in the same species. On the other hand, all collinear sequences were *aaad* genes, while all *aaad*-like genes were not collinear. Interestingly, we found that the synteny genes were not conserved between the tetrapods and teleosts (Figure 4). It seems that *aaad* family genes experienced rearrangement from teleosts to tetrapods.

### 3.2. Adaptive Evolution of AAADs in Vertebrates

Light stimulation is an important factor for maintaining physiological balance of vertebrates. Many organs take part in receiving external light signals. In all vertebrates, retinae play key photoreceptive roles in light input and reception. Lower vertebrates, like fish, can also detect light changes by pineal gland or hypothalamic suprachiasmatic nuclei (SCN) [34]. In general, the retina and pineal gland are the main organs occupying circadian organization in vertebrates. In vertebrates, melatonin is the main output of the circadian clock to convey external rhythmic information and central oscillator activity to the organism [1].

According to our results of phylogenetic analysis and protein structural comparison (Figure 4, Figure 5 and Figure 6), we observed that *aaad* genes in some cavefishes, like Mexican tetra and Sa, may be transformed into pseudogenes. These two species are cave-restricted without external light to stimulate melatonin synthesis. On the other hand, retinae can synthesize melatonin; however, cavefishes have adapted to perpetual darkness with eye degeneration and pigment loss [35,36]. Moreover, melatonin synthesis needs a prerequisition of either synthesis or intake of serotonin. Previous investigations provided evidence for uptake of serotonin by retinal photoreceptors [37,38]. However, cavefish eyes have been degenerated with the apoptosis of the lens, and they could not uptake serotonin by photoreceptors. Furthermore, in the pathway of melatonin synthesis, serotonin (5-HT) needs AAAD to realize the conversion from 5-HTP (Figure 1), while in the cave-restricted Mexican tetra and Sa, *aaad* gene may have been changed into pseudogenes, which could not convert 5-HTP into 5-HT. Since the pathway of melatonin synthesis may be blocked, we predict that rhythms in these cavefishes are weakening or disappearing. For a cave-restricted environment, energy conservation may be the major proposed driver [35] to activate selection pressure for the pseudogenization of *aaad* genes. 

Moreover, temperature is another key external clue to shape the internal rhythms beyond the alternation of light and darkness. Fishes are ectotherms and they are easily affected by external water temperature, which further influences the basis of daily and seasonal fluctuations. Many studies have reported that temperature has direct effects on the regulation of melatonin synthesis and the secretion in the pineal gland and retinae, through regulating the activity of AAANT2 [39,40,41], which is a rate-limiting enzyme for melatonin biosynthesis. Based on our results that *aaad* genes also have changed into pseudogenes in Atlantic cod, which is distributed in the North Atlantic and Arctic waters and survives in a cold water within a range from 0 to 15 °C, we suppose that AAAD activity in Atlantic cod may also be influenced by its environmental temperature. Meanwhile, Atlantic cod usually live in the deep sea of 600 meters under the water surface [42] and lack light stimulation; we, thus, speculate that *aaad* pseudogenization may have evolved to adapt well to this deep-sea and cold environment. In summary, our present study provides supportive evidence for the important role of AAAD in melatonin synthesis.

## 4. Materials and Methods

### 4.1. Gene Collection and Transcriptome Confirmation

In total, we chose 77 vertebrate genomes with relatively high-quality assembled sequences. All of them were downloaded from National Center for Biotechnology Information (NCBI), with the exception of Minnoue (*Anabarilius graham*), which was assembled by our lab (Table S1). Each genome was initially used for the construction of a standard aligned database, and was subsequently aligned by BLAST (version 2.2.28, National Center for Biotechnology Information, Bethesda, MD, USA) [43] with an E-value of 10^−5^ and employment of the protein-nucleotide aligned strategy using the AAAD proteins of Chinese alligator (*Alligator sinensis*, XP_006025391.1) as the queries. The alignment results were further processed by a Perl script to obtain the best hit of each alignment. Finally, Exonerate software (version 2.2.0, European Bioinformatics Institute, Heidelberg, Germany) [44] was employed to predict the full length of *aaad* genes. The extracted *aaad* family genes were also supported by the transcriptome evidence from liver, gill, skin, and muscle of a golden-line barbel fish (Sr) and two representative mudskipper species (BP and PM). Related transcriptome assemblies were generated in our previous studies [35,45].

### 4.2. Phylogenetic Construction and Structure Differences between AAAD Proteins

Phylogenetic analysis was performed with these predicted encoding sequences of AAADs. Multiple codon-based alignments were conducted using MEGA (version 7.0, Temple University, Philadelphia, PA, USA) [46] with the Muscle module, and each alignment of genes was manually adjusted. Subsequently, we predicted their best nucleotide substitution model using jModeltest (version 2.0, University of Vigo, Galicia, Spain) under Akaike information criterion (AIC). The parameters within the best nucleotide substitution model of GTR+I+G were applied into PhyML (version 3.1, University of Montpellier, Montpellier, France) [47,48] to construct phylogenetic topologies with the method of maximum likelihood (ML) and 1000 replicates for the evaluation of their branch supports.

To further analyze AAAD protein structures, we downloaded a common human AAAD protein model (PDB ID: 3RBF) from the Protein Data Bank (PDB). Representative sequences from medaka (*Oryzias latipes*), Chinese alligator, green anole (*Anolis carolinensis*), zebra finch (*Taeniopygia guttata*), turkey (*Meleagris gallopavo*), and tropical clawed frog (*Xenopus tropicalis*) were chosen to align with the human AAAD protein model. TEXshade [49] was applied to colorize the alignment results.

### 4.3. Conserved Synteny Identification and Phylogenetic Construction

To evaluate the conservation of *aaad* genes, we investigated several genes residing in the upstream and the downstream sequences within tetrapod and teleosts genomes through Ensembl, respectively. Finally, four genes (*IKZF1*, *FIGNL1, AAAD, Grb10*) were identified in most tetrapods, in which *aaad* genes were reported in Ensembl. In teleosts, three genes (*entpd3*, *aaad*, *GRB10b*) were conserved. Four genes (*IKZF1*, *FIGNL1, aaad, Grb10*) of human and turkey were used as the reference sequences for mammals and Aves, respectively; sequences of these four genes in Chinese alligator and tropical clawed frog were downloaded as queries for reptiles and amphibians, respectively; for teleosts, three genes (*entpd3*, *aaad*, *Grb10b*) of Platyfish (*Xiphophorus maculatus*) were used as the reference for searching syntenic locations. We obtained related genome data from NCBI and our lab as mentioned above. Subsequently, the strategy of protein aligned to nucleotides was employed to examine these extracted synteny genes in tetrapods and teleosts, respectively. Genome assemblies of different species were searched using BLAST and the best hit was selected using a Perl script.

Meanwhile, to investigate evolution of *aaad* gene in vertebrates, all the synteny sequences of *aaad* gene were further extracted for the construction of a phylogenetic tree with GTR+I+G as the best nucleotide substitution model.

### 4.4. Pseudogene Identification and Prediction of 3D Protein Structures

Combining the results of phylogenetic analysis with synteny identification, we focused on several representative species with special living habitats and important evolutionary nodes, including zebrafish, medaka, fugu (*Takifugu rubripes*), spotter gar (*Lepisosteus oculatus*), BP, PM, Atlantic cod, Mexican tetra, and three *Sinocyclocheilus* species (cave-restricted Sa, semi-cave-dwelling Sr, and surface-dwelling Sg). We implemented a multiple-sequence alignment in MEGA (version 7.0) with the Muscle module between examined species and zebrafish. Codon-based alignment was performed to cover the whole open reading frame (ORF), and the irregular ORF shifts for possible pseudogenes were identified. Missing exon region(s), codon frameshift(s), or premature stop codon(s) within each gene were considered as potential pseudogenization.

To visualize the possible variations between the standard AAAD protein of Zebrafish and mutated AAAD sequences of Atlantic cod, Mexican tetra, and Sa, we applied SWISS-MODEL (http://swissmodel.expasy.org), an automated comparative protein homology modeling, to predict corresponding 3D protein structures. Since the 3D structure of human AAAD protein had been reported (PDB ID: 3RBF), we employed it as the template for structural comparison. Finally, we visualized the predicted AAAD protein structures in PyMOL [50], and adjusted them in a similar perspective to identify variations.

### 4.5. Substitution Calculations from the AAAD Coding Sequence

To understand AAAD substitution changes in tetrapods, we chose six representative organisms, including American alligator, zebra finch, platypus, human, minke whale, and house mouse, to compare their substitution rate changes within AAAD encoding sequences. These sequences formed, in total, fifteen pairs according to pairwise combination, and we calculated the *Ka*, *Ks*, and *Ka*/*Ks* within each pair of them using DnaSP (version 5.0, University of Barcelona, Barcelona, Spain) [51].

## 5. Conclusions

In this study, we provided novel insights into *aaad* family genes in vertebrates through a genomic survey. By aligning AAAD and AAAD-like protein sequences, we observed that copy number variations of *aaad* genes in vertebrate genomes were connected with whole genome duplication and gene loss. We also revealed many similarities and differences between AAAD and AAAD-like by phylogenetic reconstruction and protein structure comparison. Interestingly, *aaad* genes in some special fishes, like cavefishes (Mexican tetra and Sa), had changed into possible pseudogenes. This phenomenon was also observed in the deep-sea Atlantic cod. Comparison of AAAD structures in Atlantic cod, and Mexican tetra and Sa further confirmed a lack of an N-terminal in Atlantic cod and a C-terminal shortage in cavefishes. We speculate that these changes may be related to the cave-restricted or deep-sea environment, and, hence, predict that the pathway of melatonin synthesis may have been blocked in part for weakening or disappearing rhythms in these fishes.

## Figures and Tables

**Table 1:** Distribution of the AAAD family in the selected vertebrate genomes.

Class	Species Number	AAAD	AAAD-Like	Total Number
Mammals	10	10	9	19
Aves	23	23	23	46
Reptiles	10	10	9	19
Amphibians	2	2	2	4
Teleosts	32	36	27	63
Total	77	81	70	151

## Supplementary Materials

The following materials are available online. Table S1. Genebank ID of the selected 77 vertebrate genomes. Table S2. Copy number variations of *aaad* genes in selected vertebrate genomes. Table S3. Fifteen pairs of combination of non-synonymous and synonymous substitutions (*Ka*/*Ks*) estimated based on *aaad* genes from six tetrapod species.
