# Supplementary material for: A Comparative Genomic Survey Provides Novel Insights into Molecular Evolution of l-Aromatic Amino Acid Decarboxylase in Vertebrates
Source: Molecules. 2018 Apr 16;23(4):917. doi: 10.3390/molecules23040917 (PMC6017361; doi:10.3390/molecules23040917)
Supplement: Supplementary file 1 [file molecules-23-00917-s001.zip › Table S1.docx]

**Table S1.** Genebank ID of the selected 77 vertebrate genomes.

| **Class** | **Common Name** | **Species Name** | **Genebank ID** |
| --- | --- | --- | --- |
| Mammals | American beaver | *Castor canadensis* | GCA_001984765.1 |
|  | Chinese tree shrew | *Tupaia chinensis* | GCA_000334495.1 |
|  | Crab eating macaque | *Macaca fascicularis* | GCA_000364345.1 |
|  | Giant panda | *Ailuropoda melanoleuca* | GCA_000004335.2 |
|  | House mouse | *Mus musculus* | GCA_000001635.25 |
|  | Human | *Homo sapiens* | GCA_000001405.37 |
|  | Minke whale | *Balaenoptera acutorostrata* | GCA_000493695.1 |
|  | Norway rat | *Rattus norvegicus* | GCA_000001895.5 |
|  | Platypus | *Ornithorhynchus anatinus* | GCA_000002275.2 |
|  | Pygmy chimpanzee | *Pan paniscus* | GCA_000258655.2 |
| Aves | Adelie penguin | *Pygoscelis adeliae* | GCA_000699105.1 |
|  | African ostrich | *Struthio camelus* | GCA_000698965.1 |
|  | American crow | *Corvus brachyrhynchos* | GCA_000691975.1 |
|  | Anna's hummingbird | *Calypte anna* | GCA_000699085.1 |
|  | Bald eagle | *Haliaeetus leucocephalus* | GCA_000737465.1 |
|  | Budgerigar | *Melopsittacus undulatus* | GCA_000238935.1 |
|  | Chimney swift | *Chaetura pelagica* | GCA_000747805.1 |
|  | Common cuckoo | *Cuculus canorus* | GCA_000709325.1 |
|  | Downy woodpecke | *Picoides pubescens* | GCA_000699005.1 |
|  | Emperor penguin | *Aptenodytes forsteri* | GCA_000699145.1 |
|  | Chicken | *Gallus gallus* | GCA_000002315.4 |
|  | Golden-collared manakin | *Manacus vitellinus* | GCA_001715985.1 |
|  | Tibetan ground-tit | *Pseudopodoces humilis* | GCA_000331425.1 |
|  | Hoazin | *Opisthocomus hoazin* | GCA_000692075.1 |
|  | Killdeer | *Charadrius vociferus* | GCA_000708025.1 |
|  | Little egret | *Egretta garzetta* | GCA_000687185.1 |
|  | Mallard | *Anas platyrhynchos* | GCA_000355885.1 |
|  | Medium ground finch | *Geospiza fortis* | GCA_000277835.1 |
|  | Peregrine falcon | *Falco peregrinus* | GCA_000337955.1 |
|  | Rock pigeon | *Columba livia* | GCA_000337935.1 |
|  | Turkey | *Meleagris gallopavo* | GCA_000146605.2 |
|  | White-throated tinamou | *Tinamus guttatus* | GCA_000705375.1 |
|  | Zebra finch | *Taeniopygia guttata* | GCA_000151805.1 |
| Reptilia | American alligator | *Alligator mississippiensis* | GCA_000281125.3 |
|  | Australian crocodile | *Crocodylus porosus* | GCA_001723895.1 |
|  | Burmese python | *Python bivittatus* | GCA_000186305.1 |
|  | Chinese alligator | *Alligator sinensis* | GCA_000455745.1 |
|  | Chinese turtle | *Pelodiscus sinensis* | GCA_000230535.1 |
|  | Garter snake | *Thamnophis sirtalis* | GCA_001077635.1 |
|  | Gharial | *Gavialis gangeticus* | GCA_001723915.1 |
|  | Green anole | *Anolis carolinensis* | GCA_000090745.1 |

**Table S1.** Continued.

| **Class** | **Common Name** | | **Species Name** | | **Genebank ID** | |
| --- | --- | --- | --- | --- | --- | --- |
|  | | Green sea turtle | | *Chelonia mydas* | | GCA_000344595.1 |
|  | | Painted turtle | | *Chrysemys picta* | | GCA_000090745.1 |
| Amphibia | | Tropical clawed frog | | *Xenopus tropicalis* | | GCA_000004195.3 |
|  | | Xizang plateau frog | | *Nanorana parkeri* | | GCA_000935625.1 |
| Teleosts | | Amazon molly | | *Poecilia formosa* | | GCA_000485575.1 |
|  | | Asian arowana | | *Scleropages formosus* | | GCA_001624265.1 |
|  | | Atlantic cod | | *Guadus morhua* | | GCA_000231765.1 |
|  | | Atlantic salmon | | *Salmo salar* | | GCA_000233375.4 |
|  | | Barramundi perch | | *Lates calcarifer* | | GCA_001640805.1 |
|  | | Blue spotted mudskipper | | *Boleophthalmus pectinirostris* | | GCA_000788275.1 |
|  | | Brichardi cichlid | | *Neolamprologus brichardi* | | GCA_000239395.1 |
|  | | Burton's mouthbrooder | | *Astatotilapia burtoni* | | GCA_000239415.1 |
|  | | Channel catfish | | *Ictalurus punctatus* | | [GCA_001660625.1](https://www.ncbi.nlm.nih.gov/assembly/763271) |
|  | | European seabass | | *Dicentrarchus labrax* | | GCA_000689215.1 |
|  | | Fugu | | *Takifugu rubripes* | | GCA_000180615.1 |
|  | | Giant fin mudskipper | | *Periophthalmus magnuspinnatus* | | GCA_000787105.1 |
|  | | Sa | | *Sinocyclocheilus anshuiensis* | | GCA_001515605.1 |
|  | | Large yellow croaker | | *Larimichthys crocea* | | GCA_000972845.1 |
|  | | Medaka | | *Oryzias latipes* | | GCA_000313675.1 |
|  | | Mexican tetra | | *Astyanax mexicanus* | | GCA_000372685.1 |
|  | | Minnoue | | *Anabarilius grahami* | | Unpublished |
|  | | Mummichog | | *Fundulus heteroclitus* | | GCA_000826765.1 |
|  | | Northern pike | | *Esox lucius* | | GCA_000721915.3 |
|  | | Nyerrrei cichlid | | *Pundamilia nyererei* | | GCA_000239375.1 |
|  | | Platyfish | | *Xiphophorus maculatus* | | GCA_000241075.1 |
|  | | Rainbow trout | | *Oncorhynchus mykiss* | | GCA_002163495.1 |
|  | | Red-bellied piranha | | *Pygocentrus Nattereri* | | GCA_001682695.1 |
|  | | tiger-tailed seahorse | | *Hippocampus comes* | | GCA_001891065.1 |
|  | | Sg | | *Sinocyclocheilus grahami* | | GCA_001515645 |
|  | | Spotter gar | | *Lepisosteus oculatus* | | GCA_000242695.1 |
|  | | Sr | | *Sinocyclocheilus rhinocerous* | | GCA_001515625 |
|  | | Stickleback | | *Gasterosteus aculeatus* | | GCA_000180675.1 |
|  | | Tilapia | | *Oreochromis niloticus* | | GCA_001858045.1 |
|  | | Tongue sole | | *Cynoglossus semilaevis* | | GCA_000523025.1 |
|  | | Zebrafish | | *Danio rerio* | | GCA_000002035.4 |
| Chondrichthyes | | Elephant shark | | *Callorhynchus milii* | | GCA_000165045.1 |
