# Supplementary material for: A Comparative Genomic Survey Provides Novel Insights into Molecular Evolution of l-Aromatic Amino Acid Decarboxylase in Vertebrates
Source: Molecules. 2018 Apr 16;23(4):917. doi: 10.3390/molecules23040917 (PMC6017361; doi:10.3390/molecules23040917)
Supplement: Supplementary file 1 [file molecules-23-00917-s001.zip › Table S2.docx]

**Table S2.** Copy number variations of *aaad* genes in the selected vertebrate genomes.

| **Class** | **Common Name** | **Species Name** | ***aaad*** | ***aaad*-like** |
| --- | --- | --- | --- | --- |
| Mammals | American beaver | *Castor canadensis* | 1 | 1 |
|  | Chinese tree shrew | *Tupaia_chinensis* | 1 | 1 |
|  | Crab eating macaque | *Macaca fascicularis* | 1 | 1 |
|  | Giant panda | *Ailuropoda melanoleuca* | 1 | 1 |
|  | House mouse | *Mus musculus* | 1 | 1 |
|  | Human | *Homo sapiens* | 1 | 1 |
|  | Minke whale | *Balaenoptera acutorostrata* | 1 | 1 |
|  | Norway rat | *Rattus norvegicus* | 1 | 1 |
|  | Platypus | *Ornithorhynchus anatinus* | 1 | - |
|  | Pygmy chimpanzee | *Pan paniscus* | 1 | 1 |
| Aves | Adelie penguin | *Pygoscelis adeliae* | 1 | 1 |
|  | African ostrich | *Struthio camelus* | 1 | 1 |
|  | American crow | *Corvus brachyrhynchos* | 1 | 1 |
|  | Anna's hummingbird | *Calypte anna* | 1 | 1 |
|  | Bald eagle | *Haliaeetus leucocephalus* | 1 | 1 |
|  | Budgerigar | *Melopsittacus undulatus* | 1 | 1 |
|  | Chimney swift | *Chaetura pelagica* | 1 | 1 |
|  | Common cuckoo | *Cuculus canorus* | 1 | 1 |
|  | Downy woodpecke | *Picoides pubescens* | 1 | 1 |
|  | Emperor penguin | *Aptenodytes forsteri* | 1 | 1 |
|  | Chicken | *Gallus gallus* | 1 | 1 |
|  | Golden-collared manakin | *Manacus vitellinus* | 1 | 1 |
|  | Tibetan ground-tit | *Pseudopodoces humilis* | 1 | 1 |
|  | Hoazin | *Opisthocomus hoazin* | 1 | 1 |
|  | Killdeer | *Charadrius vociferus* | 1 | 1 |
|  | Little egret | *Egretta garzetta* | 1 | 1 |
|  | Mallard | *Anas platyrhynchos* | 1 | 1 |
|  | Medium ground finch | *Geospiza fortis* | 1 | 1 |
|  | Peregrine falcon | *Falco peregrinus* | 1 | 1 |
|  | Rock pigeon | *Columba livia* | 1 | 1 |
|  | Turkey | *Meleagris gallopavo* | 1 | 1 |
|  | White-throated tinamou | *Tinamus guttatus* | 1 | 1 |
|  | Zebra finch | *Taeniopygia guttata* | 1 | 1 |
| Reptilia | American alligator | *Alligator mississippiensis* | 1 | 1 |
|  | Australian crocodile | *Crocodylus porosus* | 1 | 1 |
|  | Burmese python | *Python bivittatus* | 1 | 1 |
|  | Chinese alligator | *Alligator sinensis* | 1 | 1 |
|  | Chinese turtle | *Pelodiscus sinensis* | 1 | 1 |
|  | Garter snake | *Thamnophis sirtalis* | 1 | - |
|  | Gharial | *Gavialis gangeticus* | 1 | 1 |
|  | Green anole | *Anolis carolinensis* | 1 | 1 |

**Table S2.** Continued.

| **Class** | **Common Name** | **Species Name** | ***aaad*** | ***aaad*-like** |
| --- | --- | --- | --- | --- |
|  | Green sea turtle | *Chelonia mydas* | 1 | 1 |
|  | Painted turtle | *Chrysemys picta* | 1 | 1 |
| Amphibia | Tropical clawed frog | *Xenopus tropicalis* | 1 | 1 |
|  | Xizang plateau frog | *Nanorana parkeri* | 1 | 1 |
| Teleosts | Amazon molly | *Poecilia formosa* | 1 | 1 |
|  | Asian arowana | *Scleropages formosus* | 1 | 1 |
|  | Atlantic cod | *Guadus morhua* | 1 | - |
|  | Atlantic salmon^1^ | *S**almo salar* | 2 | 1 |
|  | Barramundi perch | *Lates calcarifer* | 1 | 1 |
|  | Blue spotted mudskipper^2^ | *Boleophthalmus pectinirostris* | 1 | 1 |
|  | Brichardi cichlid | *Neolamprologus brichardi* | 1 | 1 |
|  | Burton's mouthbrooder | *Astatotilapia burtoni* | 1 | 1 |
|  | Channel catfish | *Ictalurus punctatus* | 1 | 1 |
|  | European seabass | *Dicentrarchus labrax* | 1 | 1 |
|  | Fugu | *Takifugu rubripes* | 1 | 1 |
|  | Giant fin mudskipper^2^ | *Periophthalmus magnuspinnatus* | 1 | 1 |
|  | (Sa) ^1^ | *Sinocyclocheilus anshuiensis* | 1 | 1 |
|  | Large yellow croaker | *Larimichthys crocea* | 1 | 1 |
|  | Medaka | *Oryzias latipes* | 1 | 1 |
|  | Mexican tetra | *Astyanax mexicanus* | 1 | - |
|  | Minnoue | *Anabarilius grahami* | 1 | 1 |
|  | Mummichog | *Fundulus heteroclitus* | 1 | 1 |
|  | Northern pike | *Esox lucius* | 1 | 1 |
|  | Nyerrrei cichlid | *Pundamilia nyererei* | 1 | 1 |
|  | Platyfish | *Xiphophorus maculatus* | 1 | 1 |
|  | Rainbow trout^1^ | *O**ncorhynchus mykiss* | 2 | - |
|  | Red-bellied piranha | *Pygocentrus Nattereri* | 1 | - |
|  | tiger-tailed seahorse | *Hippocampus comes* | 1 | 1 |
|  | (Sg) ^1^ | *S**inocyclocheilus grahami* | 2 | 1 |
|  | Spotter gar | *Lepisosteus oculatus* | 1 | 1 |
|  | (Sr) ^1^ | *Sinocyclocheilus rhinocerous* | 2 | 1 |
|  | Stickleback | *Gasterosteus aculeatus* | 1 | 1 |
|  | Tilapia | *Oreochromis niloticus* | 1 | 1 |
|  | Tongue sole | *Cynoglossus semilaevis* | 1 | 1 |
|  | Zebrafish | *Danio rerio* | 1 | 1 |
| Chondrichthyes | Elephant shark | *Callorhynchus milii* | 1 | - |

-: unidentified; ^1^ Tetraploid fishes; ^2^ Amphibious fishes.
