# Supplementary material for: A Comparative Genomic Survey Provides Novel Insights into Molecular Evolution of l-Aromatic Amino Acid Decarboxylase in Vertebrates
Source: Molecules. 2018 Apr 16;23(4):917. doi: 10.3390/molecules23040917 (PMC6017361; doi:10.3390/molecules23040917)
Supplement: Supplementary file 1 [file molecules-23-00917-s001.zip › Table S3.docx]

**Table S3.** Fifteen pairs of combination of nonsynonymous and synonymous substitutions (*Ka*/*Ks*) estimated based on *aaad* genes from six tetrapod species.

| **Groups** | ***Ks*** | ***Ka*** | ***Ka/Ks*** |
| --- | --- | --- | --- |
| American alligator <=> Platypus | 1.43 | 0.24 | 0.17 |
| Platypus <=> Zebra finch | 1.56 | 0.24 | 0.16 |
| Human <=> Platypus | 1.70 | 0.26 | 0.15 |
| Minke whale <=> Platypus | 2.14 | 0.26 | 0.12 |
| House mouse <=> Platypus | 2.35 | 0.27 | 0.11 |
| American alligator <=> Zebra finch | 0.75 | 0.08 | 0.11 |
| Human <=> Minke whale | 0.58 | 0.05 | 0.09 |
| American alligator <=> Human | 1.83 | 0.15 | 0.08 |
| Human <=> Zebra finch | 1.74 | 0.14 | 0.08 |
| Minke whale <=> Zebra finch | 1.91 | 0.15 | 0.08 |
| House mouse <=> Minke whale | 0.81 | 0.06 | 0.08 |
| House mouse <=> Human | 0.77 | 0.06 | 0.08 |
| American alligator <=> Minke whale | 2.24 | 0.15 | 0.07 |
| House mouse <=> Zebra finch | 2.19 | 0.15 | 0.07 |
| American alligator <=> House mouse | 3.10 | 0.16 | 0.05 |
